# High-Temperature Short-Time Treatment of Human Milk for Bacterial Count Reduction

**DOI:** 10.3389/fped.2018.00359

**Published:** 2018-11-27

**Authors:** Daniel Klotz, Marie Schreiner, Valeria Falcone, Daniel Jonas, Mirjam Kunze, Andrea Weber, Hans Fuchs, Roland Hentschel

**Affiliations:** ^1^Center for Pediatrics, Department of Neonatology, Faculty of Medicine, Medical Center - University of Freiburg, Freiburg, Germany; ^2^Institute of Virology, Faculty of Medicine, Medical Center - University of Freiburg, Freiburg, Germany; ^3^Institute for Infection Prevention and Hospital Epidemiology, Faculty of Medicine, Medical Center - University of Freiburg, Freiburg, Germany; ^4^Department of Obstetrics and Gynecology, Faculty of Medicine, Medical Center - University of Freiburg, Freiburg, Germany; ^5^Institute of Medical Microbiology and Hygiene, Faculty of Medicine, Medical Center - University of Freiburg, Freiburg, Germany

**Keywords:** bacteria, human milk, cytomegalovirus, holder pasteurization, high-temperature short-time, HTST treatment, preterm

## Abstract

**Background:** Human milk (HM) for preterm infants will often be pasteurized for cytomegalovirus (CMV) inactivation and reduction of its bacterial count. High-temperature short-time (HTST) treatment compared to standard Holder pasteurization (HoP) reduces the impact of heat treatment on bioactive HM proteins while effectively inactivating CMV. No data are available for the efficacy of bacterial count reduction using HTST treatments that are available for clinical use.

**Objective:** To test the antiviral and antibacterial efficacy of HTST treatment protocols in HM using a modified HTST treatment device compared to standard HoP.

**Methods:** Holder pasteurized 95 mL HM samples were inoculated with *Staphylococcus aureus* (ATCC 6538), *Enterococcus faecalis* (ATCC 29212), *Pseudomonas aeruginosa* (ATCC 27853)*, Serratia marcescens* (Smarc 00697), two different strains of *Klebsiella pneumoniae* (ATCC 700603 and Kpn 01605) or spiked with 2 × 10^5^ 50% tissue culture infective dose of CMV (AD169) and subsequently subjected to HoP (62.5°C/30 min) or HTST treatment (62°C/5 s, 62°C/15 s, 72°C/5 s, 72°C/15 s, 87°C/2 s, and 87°C/5 s). Bacterial count was determined after treated HM was cultured for 24 h. CMV infectivity was determined by the number of specific CMV immediate early antigen stained nuclei after inoculating human fibroblasts with appropriately prepared HM samples.

**Results:** Holder pasteurized samples revealed no growth after 24 h incubation. Viable bacterial cultures were retrieved from all tested strains after HTST treatment with the default HTST protocol (62°C/5 s) that is available for clinical use. Using other time-temperature combinations, growth rates of *S. aureus, E. faecalis, P. aeruginosa, K. pneumoniae, K. pneumonia*, and *S. marcescens* were depending on treatment time, treatment temperature, bacterial genera and strain. Only after treatment temperatures above 72°C no bacterial growth was observed. CMV was inactivated by any tested time-temperature combination.

**Conclusions:** HTST treatment inactivates CMV in 95 mL HM samples but is less effective than HoP in bacterial count reduction at a time-temperature combination of 62°C/5 s. For a reliable bacterial count reduction HTST treatment at 87°C was required in this study.

## Introduction

Human milk (HM) is naturally colonized with various microbiota but its content may increase and diversify during HM handling routines in a neonatal intensive care setting (1). HM may also serve as a vector for cytomegalovirus (CMV) which is frequently reactivated in the mammary gland of the CMV seropositive lactating mother and transmitted via HM (2). HM acquired bacterial sepsis and postnatal CMV infection has been observed in preterm infants, displaying various degrees of illness from clinically unapparent infection to septicaemia and death (3). Hence, screening of maternal CMV serostatus and bacterial HM content is performed in many neonatal units (4–7). In case of suspected CMV shedding, HM for preterm infants may either be freeze-thawed or pasteurized for CMV inactivation, temporarily withheld or even discarded within some neonatal units (7). Similar strategies are pursed for bacterial HM content that is exceeding certain threshold levels or includes certain bacterial genera or species (6). Holder pasteurization (HoP, 62.5°C/30 min) which has shown to eliminate most life forms of HM microbiota will be applied by most neonatal units (6–8). However, HoP has an adverse effect on bioactive HM content and may adversely influence the immunocompetence of the preterm infant (9). In contrast, high-temperature short-time treatment (HTST, e.g., 62°C/5 s) preserves bioactive HM proteins compared to Holder pasteurization while effectively inactivating CMV (10). However, data to support the use of HTST treatment to reduce the bacterial HM content are limited and mostly generated by experimental pasteurizers that are not available for practical clinical use (10–13). The aim of this study was to test the antimicrobial efficacy of a HTST treatment system that is available for clinical use compared to standard HoP in artificially inoculated HM.

## Materials and methods

### Human milk sampling

We obtained HM samples from three mothers whose infants were treated in a German tertiary neonatal care unit, written informed consent was obtained from the donating mothers. Two of the donors were CMV seronegative as documented in their prenatal care documentation, one was CMV seropositive. Their expressed HM exceeded their infant's enteral nutrition requirements and was stored frozen in our institutional milk bank. Donors did not receive any antibiotics for at least 4 weeks prior expressing their HM.

### Human milk preparation for bacterial inoculation

HM samples from the CMV seropositive donor, frozen at −22°C, were thawed overnight at 4°C and Holder pasteurized for 30 min holding time in a water bath at a plateau temperature of 62 ± 0.5°C using a LABU Muttermilchpasteur 40 (Labu Buchrucker, Ottensheim, Austria) to eliminate any potentially colonizing HM bacteria. Immediately after the heat treatment, samples of 95 mL were prepared, and microbial cultures were performed as detailed below.

Bacteria for artificial inoculation of HM were cultured on blood agar at 36 ± 0.5°C overnight (Thermo Fisher, Waltham, MA). Thereafter colonies were suspended in 0.9% sodium chloride and enumerated using the optical density method with a turbidity meter at 620 nm (Dade Behring, Sacramento, CA). Concentration of the bacterial suspension was 1 × 10^8^ cfu/mL of which 95 μl was added to a 95 mL HM sample resulting in an inoculation dose of 1 × 10^5^ cfu/mL. Seventy-six HM samples of 95 mL were subsequently inoculated with either *Staphylococcus aureus* (ATCC 6538, *n* = 16), *Enterococcus faecalis* (ATCC 29212, *n* = 16), *Pseudomonas aeruginosa* (ATCC 27853, *n* = 12), *Klebsiella pneumoniae* (ATCC 700603, *n* = 11), *Serratia marcescens* (Smarc 00697, *n* = 12) or *Klebsiella pneumoniae* (Kpn 01605, *n* = 9). Those aliquots were then subjected to heat treatment immediately after inoculation.

The selection of tested bacterial genera and species was based on culture results from the routine HM surveillance screening of our institutional milk bank or based on clinical isolates (Smarc 00697, Kpn 01605) obtained at our neonatal unit.

### Human milk preparation for viral inoculation

**Figure 1:**
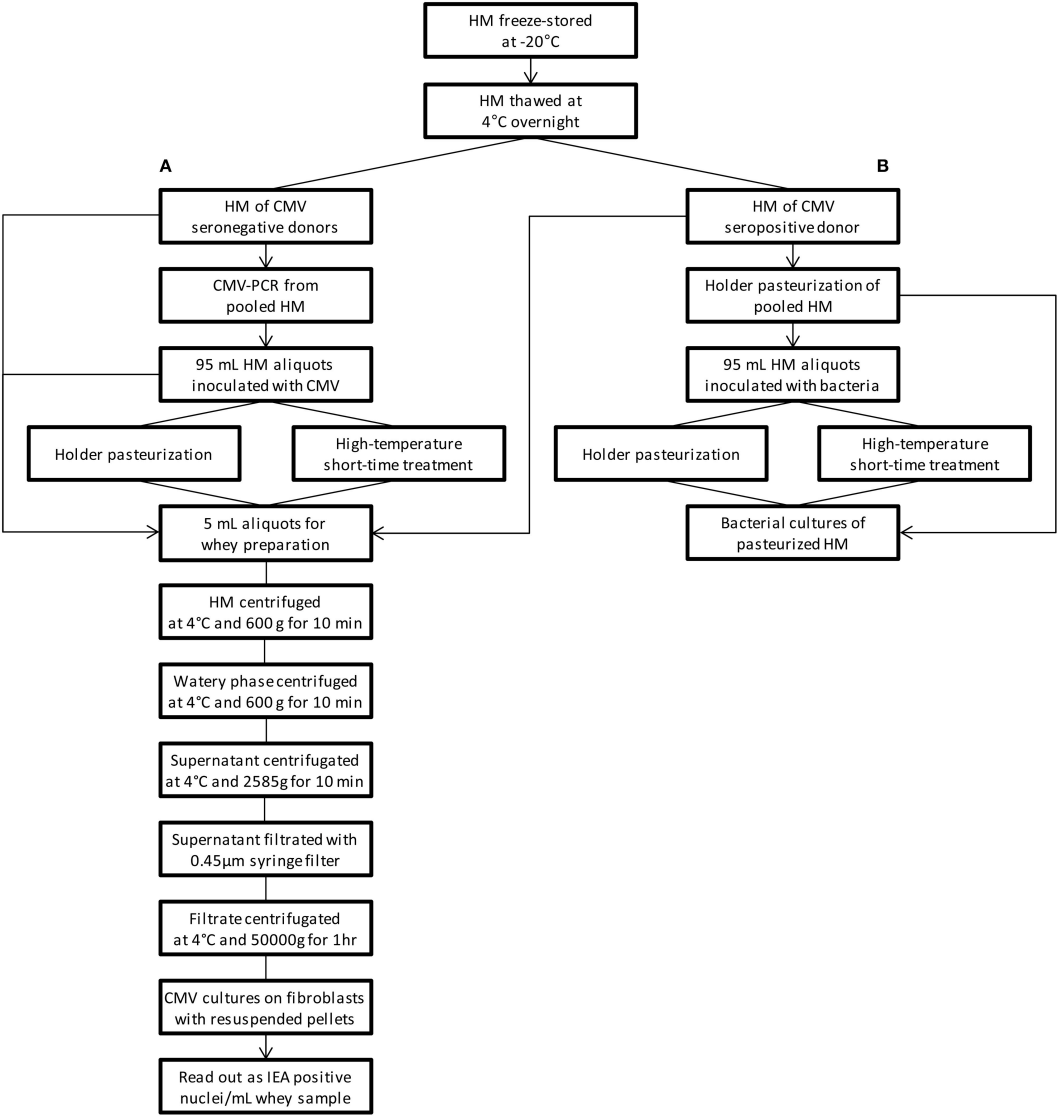
Flowchart of the study procedure. **(A)** viral studies, **(B)** bacterial studies. PCR-Studies of CMV the seropositive donor are not depicted. *HM* human milk, *CMV* cytomegalovirus, *IEA* immediate early antigen.

HM samples of the two CMV seronegative donors, frozen at −22°C, were thawed overnight at 4°C. All samples were pooled, and one unpasteurized aliquot of the pooled samples was analyzed by CMV-PCR (RealStar, altona Diagnostics, Hamburg, Germany) to confirm that donors did not excrete CMV via HM and one further unpasteurized aliquot of the CMV seropositive donor was accordingly analyzed. Sixteen aliquots of 95 mL milk were prepared and spiked with 2 × 10^5^ 50% tissue infective doses per mL/HM of cell-free culture supernatant of CMV laboratory strain AD169. An unpasteurized CMV spiked HM aliquot and an unpasteurized sample of the CMV seropositive donor served as positive reference samples for the CMV cultures. One aliquot of the seronegative donors that was not inoculated with CMV served as an unpasteurized negative reference sample for the CMV cultures. Positive and negative control samples were equally subjected to milk processing for viral analysis (Figure 1).

### High-temperature short-time treatment

High-temperature short-time treatment was performed using a Virex II (Lauf, Tübingen, Germany) as described in detail elsewhere (13). For this study this machine was modified to allow heat treatment not only at the usually non-adjustable default setting (62°C/5 s) but also at various adjustable temperatures (62, 72, and 87°C) and time (2, 5, and 15 s) combinations. To increase the applicability of HTST treatment in a human milk bank setting, the bulk volume was increased from the default volume (50 mL) to 95 mL.

The 95 mL HM samples that were inoculated with bacteria or spiked with CMV were successively placed in a rotating glass flask. The resulting thin milk layer was heated by hot air to a plateau temperature of either 62°C for a holding time of 2 s (CMV only), 5 s and 15 s or heated to 72°C for 5 and 15 s or to 87°C for 2 and 5 s (CMV not tested at 87°C), respectively (Table 1). Afterwards, the glass flask was rapidly cooled with water at 14°C.

**Table 1:** Concentration and log reduction of HM bacterial content pre and post heat treatment.

		***Staphylococcus aureus*****(ATCC 6538)**		***Enterococcus Faecalis*****(ATCC 29212)**		***Pseudomonas aeruginosa*****(ATCC 27853)**		***Klebsiella Pneumonia*****(ATCC 700603)**		***Klebsiella pneumonia*****(Kpn 01605)**		***Serratia marcescens*****(Smarc 00697)**	
		cfu/mL	Log reduction	cfu/mL	Log reduction	cfu/mL	Log reduction	cfu/mL	Log reduction	cfu/mL	Log reduction	cfu/mL	Log reduction
Pre pasteurization		7.9 × 10^4^	n.a.	1.16 × 10^5^	n.a.	1 × 10^5^	n.a.	5.7 × 10^4^	n.a.	3.6 × 10^4^	n.a.	1.04 x 10^5^	n.a.
Plateau- temperature	Plateau-time											
62.5°C	30 min	0 ± 0^#^	>4.9	0 ± 0^#^	>5.1	0 ± 0^#^	5	0 ± 0^#^	> 4.8	0 ± 0^#^	> 4.6	0 ± 0^#^	> 4.9
62°C	5 s	350 ± 150^b^	2.4	1x10^4^ ± 0^b^	1.1	7x10^3^ ± 3.6^b^	1.1	–	–	–	–	90 ± 112^d^	3.1
62°C	15 s	20 ± 14^c^	3.6	1x10^4^ ± 0^b^	1.1	20 ± 16^a^	3.7	0.5 ± 1^a^	4.5	3.6x10^3^ ± 0.5^b^	1.1	0 ± 0^#^^a^	> 4.9
72°C	5 s	40 ± 60^a^	3.3	20 ± 20^a^	3.8	0 ± 0^#^^a^	5	0 ± 0^#^^a^	>4.8	0 ± 0^#^^a^	>4.6	0 ± 0^#^^a^	> 4.9
72°C	15 s	10 ± 15^a^	3.9	20 ± 15^a^	3.8	–	–	0 ± 0^#^^a^	>4.8	0 ± 0^#^^a^	>4.6	–	–
87°C	2 s	0 ± 0^#^^a^	>4.9	0 ± 0^#^^a^	>5.1	–	–	0 ± 0^#^^a^	>4.8	–	–	–	–
87°C	5 s	0 ± 0^#^^a^	>4.9	0 ± 0^#^^a^	>5.1	0 ± 0^#^^a^	5	–	–	–	–	0 ± 0^#^^a^	> 4.9

# Not detected (lower limit of detection: < 10 cfu/mL).

Time-temperature combinations that were not analyzed were marked -

HTST treatment vs. Holder pasteurization:

a n.s,

b p < 0.0001,

c p = 0.0034,

d p = 0.002

*ATCC, American type culture collection; cfu, colony forming units; HTST, high-temperature short-time treatment; n.a., not applicable*.

Each HTST treatment for a tested time-temperature combination and for a given bacterial species as well as the CMV spiked samples was repeated two fold. Time-temperature curves of every HTST cycle were measured with a sample-frequency of 10 Hz and digitally recorded on an external hard drive. The device was cleaned according to the manufacturer's specification in between the treatments to avoid cross-contamination. Immediately after completion of the heat treatment two agar discs were inoculated with 100 μL pasteurized HM of each sample. The viral samples were stored at 4°C until further processing.

### Holder pasteurization

Three of each 95 mL HM samples that were incubated with different bacterial strains or spiked with CMV as detailed above were subjected to Holder pasteurization at 62.5 ± 0.5°C/30 min using the clinitherm Pasteur 40 (Barkey, Leopoldshöhe, Germany). Immediately after completion of the pasteurization process 100 μL of the HM samples were inoculated onto agar disks, the CMV spiked samples were stored at 4°C until further processing.

### Human milk processing for viral analysis

From each of the three Holder pasteurized CMV spiked samples, 12 HTST pasteurized CMV samples, the unpasteurized CMV spiked samples and the donors' unpasteurized positive and negative reference samples, two 5 mL aliquots (*n* = 36) were prepared using a Falcon test tube (BD, New Jersey, NJ). To separate the milk fractions aliquots were centrifuged at 4°C and 600 g for 10 min (Centrifuge 5810 R, Eppendorf, Hamburg, Germany). The resulting aqueous layer was centrifuged at 4°C and 600 g for 10 min and the subsequently resulting whey supernatant was further centrifuged at 4°C and 2585 g for 10 min. The resulting whey supernatant was filtrated with the Arodisc Syringe Filter 0,45 μm with Supor Membrane (Pall, NY, NY) and then subjected to ultracentrifugation 4°C and 50,000 g for 60 min (Optima MAX XP, Beckmann Coulter, Brea, CA). The resulting pellet was resuspended in 250 μl tissue culture medium and used to quantify CMV infectivity.

### Detection of CMV infectivity

Human foreskin fibroblasts were seeded into 24 well plates (Falcon, BD, New Jersey, NJ) and were maintained in minimal essential medium until further use (Gibco MEM supplemented with 5% fetal calf serum, Thermo Fischer). The medium was removed before inoculation and 100 μL of processed HM specimen was added to each well. Inoculated plates were centrifuged at 4°C and 900 g for 50 min (Centrifuge 5810 R, Eppendorf, Hamburg, Germany).

After centrifugation virus inoculum was removed, 1 mL of minimal essential medium was added to each well and the cultures were incubated at 37°C and 5% carbon dioxide for 18 h. Afterwards, monolayers were washed twice in phosphate-buffered saline and fixed with ice-cold acetone for 20 min. Cell monolayers were then incubated with an anti-CMV immediate early antigen (IEA) monoclonal antibody (bioMérieux, Lyon, France) and, after washing, with a fluorescein isothiocyanate-labeled goat anti-mouse immunoglobulin G conjugate (Agilent, Santa Clara, CA). Read-out was the number of CMV specific IEA stained nuclei/mL whey. Duplicates for each sample were analyzed.

### Bacterial count analysis

We inoculated Columbia blood agar with 5% sheep blood (Thermo Fisher) with 100 μL of the pasteurized whole milk samples. Duplicate plates for each sample were inoculated and counted in all instances. The agar were incubated for 24 h at 36 ± 0.5°C and 5% carbon dioxide in a Heracell incubator (Thermo Fisher) before colony-forming units per milliliter (cfu/mL) were determined with a lower limit of detection of < 10 cfu/mL.

### Data analysis and statistics

Bacterial HM concentration was determined as cfu/mL; antibacterial efficacy is reported as raw values and log reduction rates. Bacterial counts were transformed to log_10_ values for statistical analysis. A one-sample *t*-test was employed to compare the reduction in the HTST treatment with the complete reduction observed in the HoP treatment. Statistical analyses were performed using GraphPad Prism (V5.02, GraphPad, San Diego, CA). A *p*-value < 0.05 was considered significant.

## Results

**Figure 2:**
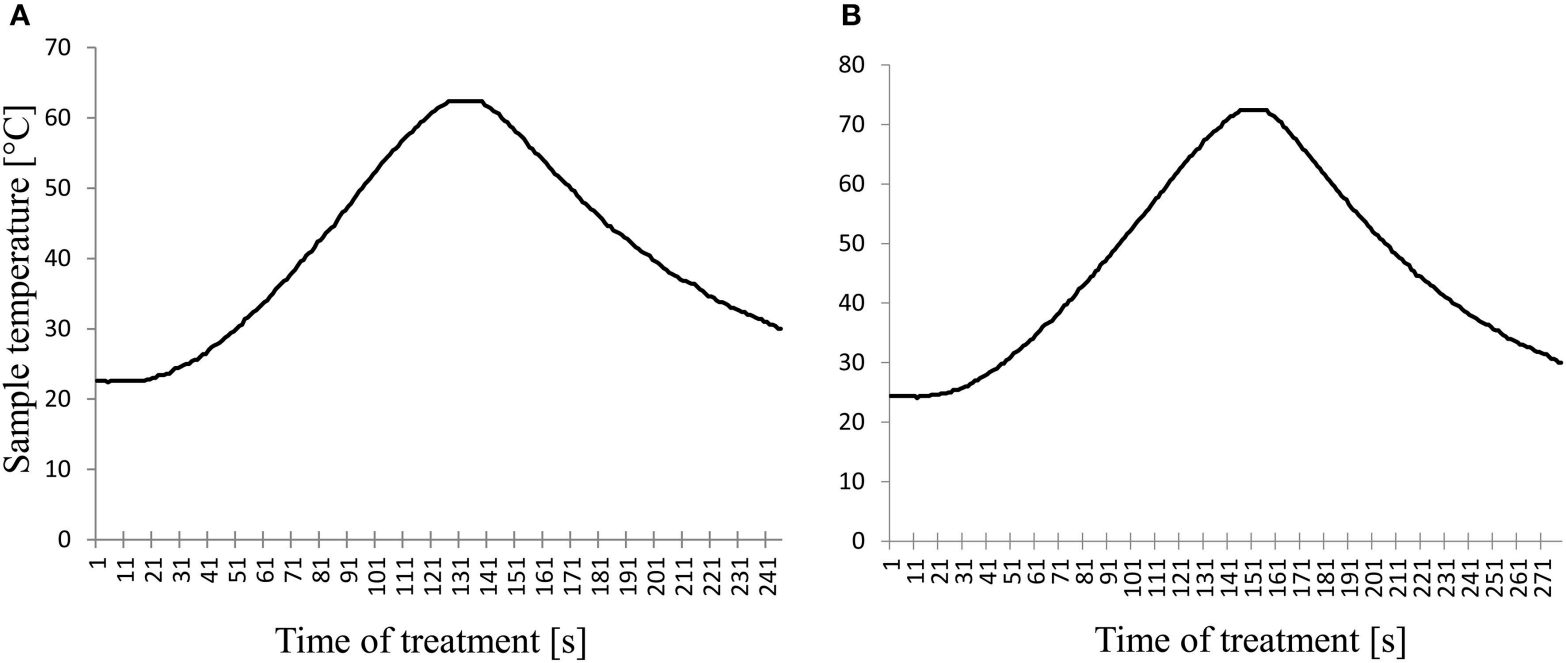
Characteristic time-temperature curves of the high-temperature short-time pasteurization processes. **(A)** time-temperature combination of 62°C/15 s; **(B)** time-temperature combination of 72°C/15 s.

A flowchart summarizing the study procedure is given in Figure 1. Characteristic time-temperature curves of the HTST pasteurization process at of 62 and 72°C/15 s are shown in Figure 2.

No bacterial growth could be detected in any of the pooled and Holder pasteurized HM samples before experimental bacterial inoculation.

Bacterial concentrations in the inoculated 95 mL HM samples after culturing for 24 h were 7.9 × 10^4^ cfu/mL for *S. aureus*, 1.16 × 10^5^ cfu/mL for *E. faecalis*, 1 × 10^5^ cfu/mL for *P. aeruginosa*, 1.04 × 10^5^ cfu/mL for *S. marcescens*, 3.6 × 10^4^ cfu/mL for *K. pneumoniae* (Kpn01605), and 5.7 × 10^4^ cfu/mL for *K. pneumoniae* (ATCC 700603). Again, following HoP no bacterial growth for any of the tested bacterial species or strains could be detected (Table 1).

Viable cultures were retrieved from all tested strains after HTST pasteurization with the default HTST protocol of 62°C/5 s of *S. aureus, E. faecalis, P. aeruginosa* (*p* < 0.0001), and *S. marcescens* (*p* = 0.002). In general, growth rates after HTST pasteurization compared to HoP depended on treatment time, treatment temperature, bacterial genera, species and strain (Table 1). Positive bacterial cultures could be retrieved for any tested plateau temperature of < 87°C for the cultures incubated with *S. aureus* and *E. faecalis* albeit differences compared to HoP were not consistently statically significant (Table 1). The samples incubated with *P. aeruginosa, K. pneumoniae* and *S. marcescens* remained without detectable growth after pasteurization of at least 72°C/5 s. The two tested strains of Klebsiella did exhibit differing heat susceptibility (*p* = 0.029). All positive cultures revealed monomicrobial growth consisting of the respective incubated strain.

Number of CMV copies obtained by PCR from the CMV seropositive donors' milk was 7,000 IE/mL and mean (SD) number of IEA positive cells in the positive control sample from the CMV seropositive donor was 20 ± 0/mL. No CMV copies were obtained by PCR and no IEA positive cells could be detected in the negative control samples from the CMV seronegative donor. After experimental CMV spiking a mean (SD) of 32 ± 5.6 IEA positive cells/mL were detected before pasteurization. CMV was inactivated by HoP and HTST pasteurization at all tested time-temperature combinations, no IEA positive cells were found.

## Discussion

This HTST treatment system was not as effective in bacterial count reduction as standard HoP at a time-temperature combination of 62°C/5 s. Colony counts of *E. faecalis* and *P. aeruginos*a were still present in abundance and counts of *S. aureus* and *S. marcescens* were reduced by more than two orders of magnitude but were still cultivable after exposure to 62°C/5 s. Only after an being exposed to 87°C all culture remained without growth.

Antibacterial efficacy of different types of HTST pasteurizers have been tested before at temperatures above 70°C and the choice of plateau temperatures within our study was modeled on these observations. Bacterial counts of naturally colonizing HM treated at 72 and 87°C for 1, 3, and 15 s was reduced by 83% to more than 99% (cfu/mL), respectively (12). Heating HM to 71°C/5.75 s, 71°C/9 s or 71°C/18.5 s in a continuous flow pasteurizer revealed more than 5 log reduction in HM artificially inoculated with *S. aureus* or *E. coli*, respectively (11). These observations are in line with our results, while temperatures as low as 62°C for bacterial count reduction in HM were not tested before.

The tested microbiota exhibited variable susceptibility to heat treatment, even between strains (8). This may explain the divergent results from previous studies where HTST treatment at 62°C/5 s of naturally colonized human milk was nearly as effective as HoP (10). Furthermore, this may result in less predictable pasteurization results if bacterial count reduction is pursued.

HTST treatment is currently applied in some neonatal units for CMV inactivation and bacterial count reduction while applying widely differing cut off values for HM microbiota content indicating pasteurization (4–7). Neonatologists that are intending to reduce bacterial HM content need to be aware of the potential limitation of HTST treatment systems in regard of their antibacterial efficacy compared to HoP. However, bacterial HM count reduction to decrease morbidity in preterm infants–namely from late-onset-sepsis and necrotising enterocolitis–remains debated because sound data to prove a beneficial influence of bacterial count reduction by pasteurization are missing (14–16).

Furthermore, any kind of heat treatment indiscriminately degrades cellular and non-cellular content of HM to a variable degree (17). Affected are both naturally colonizing HM bacteria and biologically active components that are involved in gut maturation and digestion, in shaping the infants immune system as well as its intestinal microbiome (18).

HTST has been shown to increase the retention rate of immunoglobulin A (10), lactoferrin (12, 19), and growth factors (20) amongst other proteins (21) compared to HoP. Therefore, HTST treatment as opposed to HoP of milk for preterm infants may be beneficial to improve neonatal outcome but clinical data are lacking so far. Nevertheless, HM should be subjected to the lowest energy intake possible to achieve a pursued aim, i.e., reducing bacterial count, inactivating virus or both.

CMV inactivation in HM for preterm infants is performed in many neonatal units to prevent HM acquired postnatal CMV infection (5–7). The original purpose of this tested HTST system with a non-adjustable default setting of 62°C/5 s is to inactivate CMV (13, 20). Viral inactivation is facilitated by rapidly heating a thin layer of HM within a rotating glass flask. We increased the default bulk of HM from 50 to 95 mL to advance the feasibility of HTST pasteurization in a human milk bank setting. Because of the resulting increase in thickness of the resulting fluid layer CMV inactivation had to be retested under this condition resulting in an effective CMV inactivation in 95 mL batches.

The apparent desire by some neonatologists to control bacterial content of HM should be considered when developing HM pasteurizers (22). Temperature-time combinations and modes of operation should be tested for its antibacterial efficacy (21) and guided by official regulations concerning pasteurization requirements (23). However, if exclusively CMV inactivation is pursued, a much lower energy intake may be used to treat HM and those requirements may not fit to the current definition of pasteurization of non-human milk (24).

Our study has some limitations. HTST pasteurizers utilized in previous studies were experimental prototypes with different modes of action (continuous flow vs. bulk pasteurization) and different modes of heating (plate heater exchanger vs. heating thermostat), this must be considered when comparing our findings to previous observations. We were not able to test all time-temperature combinations for every bacterium due to limited HM supply. Genera, species and strains other than those tested in our study may be present in HM. Heat susceptibility of those microbiota may be different than the ones tested, and the choice of different strains might have influenced our results. Within the study we exclusively tested HM inoculated with single strains of bacteria, but clinical HM isolates may contain multiple strains. We can only speculate if inoculation with multiple strains could have influenced our results. As different cultural agars exhibit varying recovery rates of heat-processed bacteria the choice of agar might have influenced our results (25). We chose to apply those agars used in the routine HM screening program of our neonatal unit. We did not test HM samples for the presence of bacterial growth inhibitors. Furthermore, different HM concentrations of antimicrobial proteins e.g., lactoferrin or immunoglobulins between donors could have influenced our results. However, there was no history of recent antibiotic treatment of donors and since HM was pooled results for both modes of pasteurization would have been equally affected.

## Conclusions

This HTST treatment procedure inactivates CMV in 95 mL HM samples but is not as effective as standard HoP in bacterial count reduction at a time-temperature combination of 62°C/5 s.

## Ethics statement

This study was approved by the ethics committee of the Albert-Ludwigs-University of Freiburg, Germany (No. 184/15).

## Author contributions

DK conceived and designed the study, participated in the acquisition of the HM samples, performed the pasteurization process, analyzed the data, and wrote the first draft of the manuscript. MS designed the study, performed the pasteurization process, and contributed to drafting the manuscript. VF designed the study, performed the viral assays, and contributed to data analysis and interpretation. DJ designed the study, supervised the bacterial studies, and contributed to data analysis. MK contributed to acquisition of the HM samples and drafting of the manuscript. AW contributed to the study design and reviewed the manuscript. HF and RH contributed to the study design and drafting of the manuscript. All authors reviewed the manuscript.

### Conflict of interest statement

The authors declare that the research was conducted in the absence of any commercial or financial relationships that could be construed as a potential conflict of interest.

## Abbreviations

ATCC: American type culture collection
HM: human milk
cfu: colony-forming unit
CMV: Cytomegalovirus
HoP: Holder pasteurization
HTST: High-temperature short-time
PCR: polymerase chain reaction
